# Product microbial disinfection and biotechnology: An annotated selection of World Wide Web sites relevant to the topics in *microbial biotechnology*

**DOI:** 10.1111/1751-7915.12305

**Published:** 2015-06-15

**Authors:** Lawrence P Wackett

**Affiliations:** Department of Biochemistry, Molecular Biology and Biophysics, BioTechnology Institute, University of MinnesotaSt. Paul, MN, 55108, USA

## Control of microbial growth

### http://classes.midlandstech.edu/carterp/Courses/bio225/chap07/lecture5.htm

This set of class lecture notes provides a good list and description of widely used methods for controlling microbial populations in water, foods and on surfaces.

## Methods of microbial control

### http://academic.pgcc.edu/~kroberts/web/recit/rec12.htm

This class site gives a general overview of microbial control methods.

## Control of microbial growth

### http://textbookofbacteriology.net/control_3.html

This online textbook covers all of the standard methods for killing and controlling microbes.

## Chlorination concepts: World Health Organization

### http://www.who.int/water_sanitation_health/hygiene/emergencies/fs2_17.pdf

Chlorine is one of the standard methods for water disinfection, and this Web brochure provides excellent information on how chlorine works and details of its spectrum of action.

## Water disinfection: Lenntech

### http://www.lenntech.com/processes/disinfection/disinfection.htm

This commercial site provides links to information on many different types of water disinfection, comparing and contrasting different methods.

## Methods and devices using cyanuric acid hydrolase: patent

### http://www.google.com/patents/US8367389

This patent relates to a method of enzymatically removing cyanuric acid from waters in which it builds up as a disinfection by-product and diminishes the disinfecting action of chlorine (hypochlorite).

## Microbial control in oil and gas industry

### http://www.halliburton.com/en-US/ps/multi-chem/production-challenges/Biocides.page?node-id=hsx1kyjr

Biocides are very important in the oil and gas industry to prevent fouling and souring of gas (reduction of sulfate to hydrogen sulfide). This is especially important with the advent of hydraulic fracturing in which large amounts of water are injected into shale for producing oil and gas.

## Antibacterial surfaces: review

### http://www.ncbi.nlm.nih.gov/pubmed/23434154

There are many ways to prepare surfaces to prevent the adherence and growth of microorganisms. A new field of study in this area is on the surface nanotopography of natural surfaces that are antibacterial, and this is addressed in the review article.

## Antimicrobial surface: Wikipedia

### http://en.wikipedia.org/wiki/Antimicrobial_surface

This website deals with a large number of antimicrobial surfaces with a major focus on the types of materials employed.

## UV disinfection is potentially not bacteriocidal

### http://www.nanowerk.com/news2/biotech/newsid=38865.php

This news article refers to a paper published in *Environmental Science and Technology* that suggests some bacteria become dormant upon treatment with ozone and can subsequently revive and proliferate.

## Balance between disinfection and generating disinfection by-products

### http://nepis.epa.gov/Adobe/PDF/30002I5A.pdf

This report contains much detail and describes the balance between introducing chemicals into water to kill bacteria but simultaneously avoiding chemical production of disinfection by-products that are injurious to human health.

## Disinfection products can react with personal care products

### http://www.sciencedaily.com/releases/2015/01/150106102718.htm

The chlorine that is used to kill bacteria, viruses and protozoa can also react with chemicals introduced into pools by humans; for example, the insect repellent DEET (N,N-diethyl-meta-toluamide) can react to make potentially harmful products.

## Bacterial treatments for skin

### http://www.nytimes.com/2014/05/25/magazine/my-no-soap-no-shampoo-bacteria-rich-hygiene-experiment.html?_r=0

With the advent of microbiome studies, some are considering that the skin is best cleaned by adding microbes rather than deleting them, and products are emerging to introduce desirable microbes onto the skin.

## Sewage water treatment: MicrobeWiki

### https://microbewiki.kenyon.edu/index.php/Sewage_Water_Treatment_Vat

As discussed in this wiki, wastewater treatment is largely a microbial process, but it is desirable to kill off dangerous bacteria and viruses.

## Phage therapy: Wikipedia

### http://en.wikipedia.org/wiki/Phage_therapy

This Wikipedia entry deals with the use of bacteriophages to kill off bacteria that are involved in causing human infections.

## Bacteria-killing phages

### http://discovermagazine.com/2014/march/9-bacteria-killing-phages-could-be-an-alternative-to-antibiotics

This news article discusses the use of specific bacteriophages to treat specific bacterial infections.

## Metallic copper as an antimicrobial surface

### http://www.ncbi.nlm.nih.gov/pmc/articles/PMC3067274/

This review deals with the use of copper on surfaces to kill bacteria. This is used in clinical, household and business settings.

